# Quality of care for children with acute respiratory infections in health facilities: a comparative analysis of assessment tools

**DOI:** 10.7189/jogh.12.10003

**Published:** 2022-03-26

**Authors:** Alicia Quach, Shidan Tosif, Stephen M Graham, Claire von Mollendorf, Kim Mulholland, Hamish Graham, Trevor Duke, Fiona M Russell, ARI Review group, Amy Gray, Amanda Gwee, Maeve Hume-Nixon, Saniya Kazi, Priya Kevat, Eleanor Neal, Cattram Nguyen, Rita Reyburn, Kathleen Ryan, Patrick Walker, Chris Wilkes, Yasir Bin Nisar, Jonathon Simon, Wilson Were

**Affiliations:** 1 Asia-Pacific Health Group, Murdoch Children’s Research Institute, Victoria, Australia; 2 Department of Paediatrics, The University of Melbourne, Victoria, Australia; 3 Murdoch Children’s Research Institute, Victoria, Australia; 4 The Royal Children’s Hospital, Parkville, Victoria, Australia; 5 Burnet Institute, Melbourne, Victoria, Australia; 6 London School of Hygiene and Tropical Medicine, London, UK

## Abstract

**Background:**

Severe childhood pneumonia requires treatment in hospital by trained health care workers. It is therefore important to determine if health facilities provide quality health services for children with acute respiratory infections (ARI), including pneumonia. Using established indicators from WHO to measure quality of care (QoC) as a reference standard, this review aims to evaluate how well existing tools assess QoC for children presenting to health facilities with ARI.

**Methods:**

Existing assessment tools identified from a published systematic literature review that evaluated QoC assessment tools for children (<15 years) in health facilities for all health conditions were included in this ARI-specific review. 27 ARI-specific indicators or “quality measures” from the WHO “Standards for improving quality of care for children and young adolescents in health facilities” were selected for use as a reference standard to assess QoC for children presenting to health facilities with ARI symptoms. Each included assessment tool was evaluated independently by two paediatricians to determine how many of the WHO ARI quality measures were assessable by the tool. The assessment tools were then ranked in order of percentage of ARI quality measures assessable.

**Results:**

Nine assessment tools that assessed QoC for children attending health facilities were included. Two hospital care tools developed by WHO had the most consistency with ARI-specific indicators, assessing 22/27 (81.5%) and 20/27 (74.1%) of the quality measures. The remaining tools were less consistent with the ARI-specific indicators, including between zero to 16 of the 27 quality measures. The most common indicators absent from the tools were assessment of appropriate use of pulse oximetry and administration of oxygen, how often oxygen supply was unavailable, and mortality rates.

**Conclusions:**

The existing WHO hospital-based QoC assessment tools are comprehensive but could be enhanced by improved data quality around oxygen availability and appropriate use of pulse oximetry and oxygen administration. Any tools, however, should be considered within broader assessments of QoC, rather than utilised in isolation. Further adaptation to local settings will improve feasibility and facilitate progress in the delivery of quality health care for children with ARI.

**Registration:**

The protocol of the original systematic review was registered in PROSPERO ID: CRD42020175652.

Child mortality is a focus of the Sustainable Development Goals (SDGs) aimed at ending preventable deaths of young children by 2030. Outside the neonatal period, pneumonia is the leading cause of under-five mortality and, at the end of 2019, was responsible for 14% of all under-five deaths [1].

The integrated Global Action Plan for the Prevention and Control of Pneumonia and Diarrhea (GAPPD), developed by the World Health Organization (WHO) and the United Nations Children’s Fund (UNICEF), aims to end preventable pneumonia and diarrhoea-related child deaths by 2025 through targeting a comprehensive package of interventions [2]. Most of these interventions are delivered at the community level. The interventions focusing on treatment of pneumonia include increasing access to appropriate health care and case management for pneumonia. The majority of childhood pneumonia cases can be managed in the community or at the first-level health facility if there is timely access to care, recognition and classification of pneumonia severity, and dispensing of appropriate antibiotics [3]. Severe pneumonia (cough or difficulty breathing, and one or more general danger signs), however, requires referral and treatment in a hospital with injectable antibiotics and other supportive measures such as supplemental oxygen [3]. All levels of health facilities, therefore, need to be adequately equipped with quality health services to ensure the best outcomes for sick children.

The WHO developed a set of quality standards and detailed measures (“Standards for improving the quality of care for children and young adolescents in health facilities”) to provide a structured approach when addressing quality of care (QoC) for children in health facilities [4]. There are eight overarching quality standards that encompass the broad categories of provision of care, experience of care, and availability of human and physical resources to meet the best interests of children. Children presenting with acute respiratory infections (ARI) or pneumonia make up a large proportion of health facility presentations. Ensuring that children receive quality health services whilst attending the health facility is vital to improving child health outcomes and reducing overall child mortality.

We recently undertook a systematic literature review to identify and evaluate existing tools used to assess the QoC for children attending health facilities for all presentations [5]. For this analysis, we aim to evaluate how comprehensively the identified tools from the published systematic review assess ARI-specific indicators outlined in the WHO “Standards for improving quality of care for children and young adolescents in health facilities”.

## METHODS

### Search strategy and selection criteria

Using PRISMA reporting guidelines [6], our original systematic review searched the literature in August 2020 to identify assessment tools used globally to assess QoC for children attending health facilities [5]. The details of the search strategy and selection process of the assessment tools are available from our recent peer-reviewed publication [5] and are provided in Appendices S1 and S2 in the **Online Supplementary Document**. In brief, a systematic literature search was performed of MEDLINE (Ovid), PubMed and Global Health (CAB direct) databases and The International Journal for Quality in Health Care using Medical Subject Heading (MeSH) terms and/or keywords. Grey literature was identified through World Bank and WHO library databases using keywords. The inclusion criteria included publications/reports that were published in the English language and reported the use of an assessment tool to evaluate quality of health care in any health care facility. The search period was limited from 2008 to 2020 to identify tools more likely to be in current use. Assessment tools were deemed eligible if used by more than one country, and if they included at least one module/component evaluating QoC in children, aged 0-14 years. Tools were excluded if they assessed only newborns (<1 month old) or only adolescents (10-19 years old). The purpose of the original systematic review was to identify the most comprehensive tools in use and available in a global context. Assessment tools that were developed only for research purposes and those that only evaluated the QoC for a specific disease, such as pneumonia; or a specific component of QoC (eg, antimicrobial prescribing practice) were excluded. The screening and selection processes to identify eligible assessment tools were performed independently by two reviewers. The protocol of the original systematic review from which the publications/reports in this review were drawn was registered in PROSPERO ID: CRD42020175652.

### Data analysis

For this ARI review, 27 measurable indicators or “Quality Measures (QMs)” were purposively selected from WHO “Standards for improving quality of care for children and young adolescents in health facilities”, with consensus amongst the authors, as specific to care for children with ARI and aligning with current WHO ARI clinical guidelines (**Table 1**) [4,7]. For each assessment tool included, modules and questions were reviewed to decide if they matched any of the 27 ARI-specific QMs. To decrease the risk of bias, two paediatricians (AQ, ST) performed the matching process independently. Any conflicts were discussed between the two reviewers. For any unresolved conflicts, a third reviewer (FMR) was consulted.

**Table 1:** Summary of WHO ARI-specific Quality Measures assessable by each quality assessment tool*

	Quality assessment tool								
	**WHO-Europe**	**WHO-SE. Asia**	**SPA**	**HeRAMS**	**r-HFA**	**SARA**	**HFS-IMCI**	**HRBF**	**HCAHPS-Child**
**ARI-specific Quality Measure**									
**Quality Measure 1.1.2 (Input)** The health facility has the essential equipment and supplies for assessing and monitoring paediatric emergencies (eg, weighing scales, thermometer, blood pressure measuring device, blood glucose and oxygen saturation tests).	Y	Y	Y	Y	Y	Y	Y	Y	N
**Quality Measure 1.2.11 (Process/Output)** Proportion of all sick young infants admitted to the facility with PSBI or fast breathing who were appropriately assessed for oxygen requirements with a pulse oximeter and received the documented appropriate amount of oxygen.	Y	Y	N	N	N	N	N	N	N
**Quality Measure 1.3.1 (Input)** The health facility has a written, up-to-date, evidence-based clinical protocol for identifying and managing children with cough or difficult breathing, consistent with IMCI and paediatric care guidelines.	Y	Y	Y	Y	Y	Y	Y	N	N
**Quality Measure 1.3.2 (Input)** The referral receiving health facility has basic laboratory and diagnostic tests (eg, pulse oximetry, full blood count, culture, ultrasound, and chest x-ray) available for appropriate investigation of children with severe pneumonia.	Y	Y	Y	Y	Y	Y	N	Y	N
**Quality Measure 1.3.3 (Input)** The health facility has adequate supplies of antibiotics (first- and second-line) for treatment of severe pneumonia and pneumonia for the expected case load with no stock outs.	Y	Y	Y	Y	Y	Y	Y	Y	N
**Quality Measure 1.3.4 (Input)** The health facility has adequate supplies of inhalation bronchodilators and delivery devices for treatment of wheeze for the expected case load with no stock outs.	Y	Y	Y	Y	N	Y	N	N	N
**Quality Measure 1.3.5 (Input)** The health facility has an adequate supply of pulse oximeters and a reliable, functioning oxygen supply at all times for the expected case load with no stock outs.	Y	Y	Y	Y	N	Y	N	Y	N
**Quality Measure 1.3.6 (Input)** The health facility clinical staff who care for children receive training and regular refresher sessions in assessing and managing children with cough or wheeze at least once every 12 months.	Y	N	Y	N	Y	N	N	N	N
**Quality Measure 1.3.7 (Process/Output)** Proportion of all children with cough or difficult breathing who are correctly assessed, investigated, classified, and diagnosed according to the severity of pneumonia.	Y	Y	Y	Y	Y	N	Y	N	N
**Quality Measure 1.3.8 (Process/Output)** Proportion of children <5 years with cough or difficult breathing treated as outpatients who were correctly classified according to IMCI guidelines.	Y	Y	Y	Y	Y	N	Y	N	N
**Quality Measure 1.3.9 (Process/Output)** Proportion of all children with pneumonia or severe pneumonia who received correct antibiotic treatment (formulation, dose, frequency and duration) according to WHO guidelines.	Y	Y	Y	Y	Y	N	Y	N	N
**Quality Measure 1.3.10 (Process/Output)** Proportion of all children with asthma who were appropriately administered inhalation bronchodilator treatment.	Y	Y	Y	Y	N	N	N	N	N
**Quality Measure 1.3.11 (Process/Output)** Proportion of all children with pneumonia to whom oxygen was appropriately administered for the clinical indication (signs of hypoxaemia or oxygen saturation <90%).	Y	Y	N	Y	N	N	N	N	N
**Quality Measure 1.3.12 (Process/Output)** Proportion of all children admitted with severe pneumonia whose respiratory rate and oxygen saturation were appropriately monitored.	Y	Y	N	Y	N	N	N	N	N
**Quality Measure 1.3.13 (Process/Output)** Proportion of all children with cough for ≥14 days who were referred or further assessed and investigated for TB or other causes of chronic infection.	Y	Y	N	N	N	N	N	N	N
**Quality Measure 1.3.14 (Process/Output)** Proportion of all children with only cough and cold (with no signs of pneumonia or severe pneumonia) who received antibiotics.	Y	N	Y	N	N	N	Y	N	N
**Quality Measure 1.3.15 (Outcome)** Proportion of all children managed for pneumonia in the health facility who died of pneumonia (case fatality rate).	N	Y	N	N	N	N	N	N	N
**Quality Measure 1.3.16 (Outcome)** Proportion all children who died of pneumonia among all children admitted to the health facility.	Y	Y	N	N	N	N	N	N	N
**Quality Measure 1.3.17 (Outcome)** Proportion of all children managed for pneumonia in the health facility who died of pneumonia within the initial 24 h of admission.	N	Y	N	N	N	N	N	N	N
**Quality Measure 1.3.18 (Outcome)** Proportion of all children managed for wheeze or asthma in the health facility who died of wheeze.	N	N	N	N	N	N	N	N	N
**Quality Measure 1.13.7 (Process/Output)** Proportion of all children who received oxygen for which the prescribed method and rate of delivery are documented.	Y	N	N	N	N	N	N	N	N
**Quality Measure 1.15.9 (Process/Output)** Proportion of children up to 5 years seen at the health facility with cough who receive harmful cough remedies for respiratory tract infections.	Y	N	Y	N	Y	N	N	N	N
**Quality Measure 8.3.4 (Input)** The health facility has a safe, uninterrupted source of oxygen and equipment for delivery (age-appropriate nasal prongs, catheters, and face masks) available at all times in children’s wards and emergency areas.	Y	Y	Y	Y	N	Y	N	N	N
**Quality Measure 8.3.5 (Input)** The health facility has basic equipment (x-ray, ultrasound, and basic laboratory equipment) for diagnosis and management of common childhood illnesses and conditions.	Y	Y	Y	Y	N	Y	N	Y	N
**Quality Measure 8.3.13 (Outcome)** Proportion of days per calendar year during which an oxygen source and delivery were not available.	N	N	N	N	N	N	N	N	N
**Quality Measure 8.4.7 (Input)** The health facility has adequate essential child-friendly equipment and medical supplies, including an oxygen source, to support routine and emergency management of children.	Y	Y	Y	Y	N	N	N	Y	N
**Quality Measure 8.4.13 (Outcome)** Proportion of days in the past 3 months when oxygen was not available in the health facility in areas in which children are cared for.	N	N	N	N	N	N	N	N	N
**Total Quality Measures assessable – n (%)**	**22 (81.5%)**	**20 (74.1%)**	**16 (59.3%)**	**15 (55.6%)**	**8 (29.6%)**	**8 (29.6%)**	**7 (25.9%)**	**6 (22.2%)**	**0 (0%)**

ARI – acute respiratory infection, WHO – World Health Organization, HeRAMS – Health Resources Availability Mapping System, SPA – Service Provision Assessment, r-HFA – rapid Health Facility Assessment, SARA – Service Availability and Readiness Assessment, HFS-IMCI – Health Facility Survey – using Integrated Management of Childhood Illness clinical guidelines, HRBF – Health Results-Based Financing impact evaluation toolkit, HCAHPS – Hospital Consumer Assessment of Healthcare Providers and Systems,

*N indicates that the assessment tool did not assess the corresponding Quality Measures, Y indicates the Quality Measure was assessable.

We used the same scoring system from the original systematic review where a question/statement from an assessment tool was considered a match to a WHO QM if any component of the QM was included. A score of 1 was allocated for a match with a maximum score of 1 for each QM, even when matched with multiple questions from an assessment tool. For QMs that consisted of more than one subcomponent, only one subcomponent had to be matched to a question/statement from the assessment tool to be allocated a score of 1. For each assessment tool, descriptive summary measures were calculated to determine the percentage of the 27 ARI-specific QMs assessable by each tool and then ranked in order of their total percentages.

## RESULTS

The original systematic review identified 1180 publications/reports with 145 full-text manuscripts assessed for eligibility. Nine unique assessment tools were extracted from 39 eligible publications/reports. The PRISMA flow diagram for the selection of assessment tools has been published with the findings from the original systematic review [5] and is also available in Appendix S2 in the **Online Supplementary Document**. All nine assessment tools identified from the original systematic review were included in this review. **Table 2** summarises the nine assessment tools, with the modules that had questions/statements matched to a WHO ARI-specific QM listed. All tools were structured questionnaires/interviews with checklist-style questions. The tools measured QoC in a range of health care settings (eg, primary health care facilities, hospitals, emergency response health facilities) with the majority developed for use in low- and middle-income countries (LMICs).

**Table 2:** Summary of assessment tools reviewed for quality of care for children in health facilities

Tool	Development/ Source	Type of health facility tool was designed for	Key objectives of the tool	Modules that assessed the ARI specific QMs
Service Provision Assessment (SPA) [8]	USAID – MEASURE Evaluation	All health facility types	Assessment of availability and service readiness of health services, adherence to standards of care and satisfaction levels of clients and service providers	• Inventory
				• Clinical observation – sick child (age not specified)
				• Health worker interview
Rapid Health Facility Assessment (r-HFA) [9]	USAID - MEASURE Evaluation	Primary health care facilities providing maternal, neonatal and child health services	Assessment of case management of common childhood illnesses, service readiness and quality of management of health facilities.	• Clinical observation – sick child (age 1-59 mo)
				• Health worker interview and record review
				• Health facility checklist
Service Availability and Readiness Assessment (SARA) [10]	WHO	All health facility types	Assessment of service availability and service readiness of health facilities	• Infrastructure
				• Available services
				• Diagnostics
				• Medicines and commodities
Health Facility Survey – using Integrated Management Childhood Illness as clinical guidelines (HFS-IMCI) [11]	WHO	Primary level health facilities providing outpatient care for sick children	Assessment of quality of care delivered to sick children attending first-level outpatient health facilities, using the Integrated Management of Childhood Illness (IMCI) clinical guidelines as best practice. Quality of care defined as case management, quality of counselling, availability of health system supports and barriers to effective integrated management.	• Clinical observation – sick child (age 2-59 mo)
				• Equipment and supply checklist
Assessment Tool for Hospital Care (WHO-SE. Asia Office) [12]	WHO – SE. Asia Office	All hospitals providing maternal, neonatal and child health services	Assessment of quality of care for maternal, neonatal and child health services in hospitals, based on standards from the WHO Pocket book of hospital care for children and WHO integrated management of pregnancy and childbirth (IMPAC).	• General hospital information
				• Newborn care
				• Paediatric care (age not specified)
				• Postal questionnaire
Hospital care for children: quality assessment and improvement tool (WHO-Europe Office) [13]	WHO – Europe Office	All hospitals providing child health services (excluding neonatal health care)	Assessment of service availability and readiness of hospital, including policies and adherence to guidelines, satisfaction levels of clients and service providers	• Hospital support services
				• Case management
				• Policies and organisation of services
Health Resources Availability Mapping System (HeRAMS) [14]	WHO	Humanitarian and Emergency Response settings	Survey to assess and monitor status of health facilities and availability of health services and resources for adults and children in areas affected by emergencies.	• Hospital assessment tool
				• Health centres assessment tool
Health Results-Based Financing (HRBF) impact evaluation toolkit [15]	World Bank	All health care facilities (Program evaluating the impact of health-related results-based financing incentives)	Multinational program evaluating the impact of health-related results-based financing incentives/ interventions (with focus on maternal and child health programs) on the access to and quality of service delivery, health expenditures and health outcomes.	• Health facility assessment
Hospital Consumer Assessment of Healthcare Providers and Systems – Child Version (HCAHPS-Child) [16]	Boston Children’s Hospital	All hospitals providing inpatient care for children	Single centre-based survey/audit to measure the patient-centredness of hospital care for paediatric patients (age 0-17 years).	• Nil

ARI – acute respiratory infection. QM – quality measure

**Figure 1:**
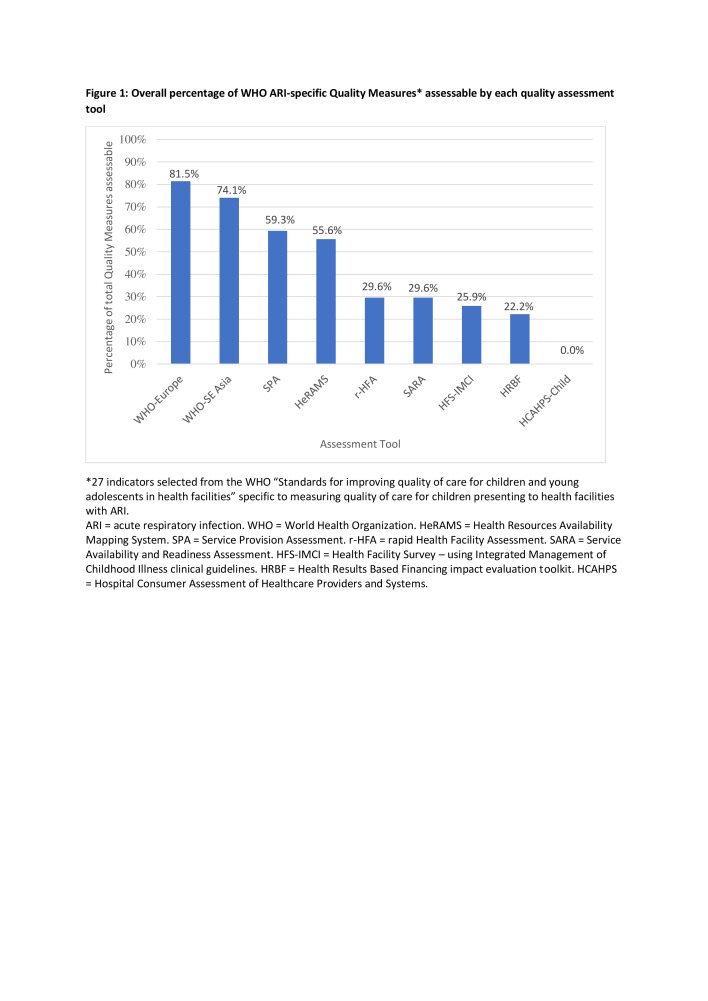
Overall percentage of WHO ARI-specific Quality Measures* assessable by each quality assessment tool. ARI – acute respiratory infection, WHO – World Health Organization, HeRAMS – Health Resources Availability Mapping System, SPA – Service Provision Assessment, r-HFA – rapid Health Facility Assessment, SARA – Service Availability and Readiness Assessment, HFS-IMCI – Health Facility Survey – using Integrated Management of Childhood Illness clinical guidelines, HRBF – Health Results-Based Financing impact evaluation toolkit, HCAHPS – Hospital Consumer Assessment of Healthcare Providers and Systems. *27 indicators selected from the WHO “Standards for improving quality of care for children and young adolescents in health facilities” specific to measuring quality of care for children presenting to health facilities with ARI.

**Figure 1** shows the overall percentage to which each tool was able to assess the 27 WHO ARI-specific QMs. The WHO (Europe regional office) “Hospital care for children: quality assessment and improvement tool” was the most comprehensive with (22/27 = 81.5%) of the ARI QMs being assessable with the tool [13]. The Child Hospital Consumer Assessment of Healthcare Providers and Systems (HCAHPS-Child) survey tool did not assess any of the ARI QMs [16]. The remaining tools assessed between six and 16 out of the 27 ARI QMs.

**Table 1** details which of the 27 ARI QMs were assessable by each tool. Of the selected 27 ARI QMs, 10 were input QMs, including the availability of essential equipment, tests, and medicines, 11 were process/output QMs, such as proportion of children who were appropriately assessed, diagnosed and received appropriate treatment for pneumonia, and 6 were outcome QMs, including mortality rates and proportion of days when there was no oxygen supply available. Tools generally captured QMs relating to input measures (median = 8/10), better than process/output (median = 4/11) and outcome (median = 0/6) QMs. Key process/output indicators relating to clinical assessment, and classification of severity of pneumonia was not assessable by three of the nine tools. Aside from the WHO Europe and WHO-SE. Asia tools, pneumonia mortality data were not routinely collected by the assessment tools.

Oxygen availability was assessable by most tools, but only three tools (WHO-Europe, WHO-SE. Asia and Health Resources Availability Mapping System (HeRAMS)) [12-14] assessed whether there was appropriate oxygen administration for severe disease, and only two of these (WHO-Europe and WHO-SE. Asia) also assessed for appropriate use of pulse oximetry for measurement of oxygen saturation. Only the WHO-Europe tool documented the rate and delivery of oxygen; and no tools collected data on how often there was a shortage of oxygen supply (QM 8.3.13, QM 8.4.13) [4].

Six out of the nine tools assessed whether appropriate antibiotics were prescribed for pneumonia, but only three tools (WHO-Europe, SPA, Health Facility Survey – Integrated Management of Childhood Illness guidelines (HFS-IMCI)) [8,11,13] assessed whether antibiotics were prescribed inappropriately for children presenting with mild cough and cold symptoms.

## DISCUSSION

This is the first comparative analysis of assessment tools for QoC for children attending health facilities against ARI-specific indicators in the WHO “Standards for improving quality of care for children and young adolescents in health facilities”. The 27 ARI-specific QM selected as reference standards for this analysis reflect the WHO ARI guidelines for hospital care for children, evaluating whether a child is clinically assessed and diagnosed according to the severity of pneumonia and whether they receive appropriate treatment with antibiotics and oxygen [7]. Mortality indicators due to ARI/pneumonia were included as important outcomes for monitoring case management and QoC for ARI/pneumonia. The Hospital Care assessment tools developed by the WHO-Europe and WHO-SE. Asia regional offices were found to be the most comprehensive for assessing QoC for children in health facilities with ARI, with three-quarters or more of the QMs included in their tools [12,13]. The remaining assessment tools included in this review did not adequately assess the ARI QMs, with five of the nine included tools covering less than one-third of the recommended indicators.

Input QMs were more readily assessable by all the assessment tools than process/output and outcome QMs. The tools included in this review were designed to give a “snapshot” analysis of the health facility, most with checklist-style questions. These make the input QMs easier and quicker to assess. The tools that were able to assess process/output QMs required access to patient records and health information systems in order to collect and analyse data over time, with the WHO Hospital Care assessment tools being the only ones to collect mortality data. Quality improvement practices for ARI therefore not only rely on comprehensive quality assessment tools to monitor progress but also on adequate record-keeping and established health information systems within the health facilities.

The WHO Pocketbook of Hospital Care for Children is a widely used resource for up-to-date evidence-based clinical guidelines for the management of sick children presenting to a hospital [7,17]. The ARI-specific QM selected were based on their alignment with the chapter on the management of a child presenting with cough. The WHO Hospital Care assessment tools have been adapted and updated to align with guidelines set out in the WHO Pocketbook, and both also include modules dedicated to case management of respiratory symptoms, which likely contributed to their closer consistencies with the ARI-specific QMs in this review.

Other tools included in this review which had different primary objectives did not assess the ARI-specific QMs to the same extent. The HCAHPS aims to capture the patient/caregiver perspectives of hospital quality of care and hence did not include any ARI-specific questions. The Service Availability and Readiness Assessment (SARA) tool focuses on input QMs to determine the “readiness” of a health facility for delivering health care, with less emphasis on how well these services are actually delivered. The Service Provision Assessment (SPA) and rapid-Health Facility Assessment (r-HFA) tools include input QMs to determine service readiness as well as process/output QM such as assessments/observations of case management, but do not have ARI specific modules, leaving out more detailed specific components of ARI management.

The setting and target age group of the assessment tool also affected how comprehensively it assessed the ARI QMs. The HFS-IMCI tool was developed for use in the primary health care/outpatient settings and hence did not include QMs associated with hospital admissions and management of severe pneumonia. The HeRAMS tool is used in humanitarian and emergency response settings, in order to determine and deliver time-critical emergency resources needed. More time-consuming data analyses requiring valuable human resources are usually not able to be completed in these settings.

Common indicators that were absent from many of the tools were related to oxygen therapy. Although most of the reviewed tools assessed whether supplementary oxygen was available, very few assessed the appropriate use of pulse oximetry, oxygen administration, or methods of oxygen delivery. The 20th edition of the WHO Model List of Essential Medicines in 2017 was updated to include oxygen for the management of hypoxaemia [18]. Appropriate oxygen administration and monitoring of oxygen saturation are cost-effective measures for the management of pneumonia, and yet access to oxygen therapy is not a current standard indicator in the integrated GAPPD [2,19]. There is a need for more high-level data on oxygen use and monitoring to ensure that access to oxygen supply is being delivered in an effective manner [20,21]. Affordable access to uninterrupted oxygen supply should be a key indicator included in any assessment of standard ARI/pneumonia management. To determine quality of care, further components including rational administration by trained staff, monitoring with functional pulse oximeters and appropriate methods of oxygen administration must also be included in the assessment.

Many health facilities in LMICs are limited in resources and must prioritise service provision accordingly. Specific quality improvement activities and priority indicators should be driven by local context, but better consensus on key indicators could help guide facilities and child health programs. Although this review focused on the QoC for ARI, it should be considered in the broader context of comprehensive QoC assessment. Having 27 QM to assess for ARI may therefore be impractical when combined amongst other quality improvement activities. The ARI-specific QM selected from the WHO Standards in this review are repetitive in some of their measures. QM 1.1.2 and QM 8.4.7 both assessed whether the health facility had an adequate supply of essential equipment and medical supplies; QM 1.3.5 and QM 8.3.4 both assessed availability of adequate oxygen supply; QM 1.3.2 and QM 8.3.5 both assessed availability of basic laboratory equipment such as x-rays. Merging or simplifying some of the indicators may make it easier for health facilities to translate and implement them into their quality improvement practices. Development of a monitoring tool that highlights key indicators to track over time may go on to inform progress at local and national levels.

Feasibility is the other important consideration for any QoC assessment activity. Having an existing, commonly used tool to assess key indicators for ARI QoC can further assist resource-limited settings in their quality improvement activities. The tool, however, needs to be affordable, simple enough to be implemented by staff with varying levels of education and training, and robust enough to collect data that can inform and affect change in practice. The assessment tools included in this review were all freely available. They were, however, variable in length and detail. Some such as the HCAHPS-Child required only binary or multi-choice responses, whereas others required clinical observation or interviews with staff and/or caregivers. The WHO Hospital tools which were most consistent with the ARI-specific QM are extensive in their current formats. It is less clear how these tools have been used in practice with little anecdotal evidence that they are widely implemented. There are examples, however, of how a single tool, when designed well to meet its purpose with appropriate feedback loops, can impact change. The Malawi Child Lung Health Program was a successful integrated public health intervention, that used the same tool to perform situational analysis, address gaps and provide feedback, and simultaneously collate evaluation and monitoring data [22]. It was implemented on a nationwide scale at the district (secondary health care) facility level and, in conjunction with steps taken to strengthen identified deficiencies in QoC, demonstrated a reduction in pneumonia case fatality rates. There needs to be further research into the feasibility of tools to assess quality of health care. Modification and simplification of existing tools with consideration of how key indicators can be integrated into wider public health interventions may make them more achievable in LMICs.

This review has limitations. In the tool evaluation process, clinical judgement was made as to whether questions/items from assessment tools adequately matched the QMs in the WHO Standards. Although two reviewers independently performed the evaluation, this was a subjective process that could have led to bias in some indicators. The nine assessment tools that were included in this review were part of a broader systematic review evaluating QoC for children at health facilities for all presentations. We, therefore, did not include tools that were evaluating QoC specifically for pneumonia/ARI. Future reviews may identify such existing tools and evaluate how consistent they are with the WHO ARI-specific QMs. The ARI-specific QMs used for this review did not incorporate all the quality standards outlined in the WHO Standards, and hence findings from this review on the comprehensiveness of the tools may not be applicable to the broader concepts of QoC.

## CONCLUSION

There are existing WHO Hospital Care assessment tools that can adequately assess ARI-specific indicators recommended in the WHO Standards. The WHO ARI QMs could be simplified by identifying key indicators to assess for optimal care for ARI. Reporting on oxygen access should include uninterrupted supply, appropriate administration, and monitoring with pulse oximetry. Assessing QoC for ARI should be considered within the broader context of QoC and any tools should be incorporated into more comprehensive quality improvement practices rather than utilised as standalone tools. Further study into the feasibility of these tools is required to ensure that they are practical for resource-limited settings, and data collected can inform local quality improvement practices in an effective manner. The development of simple monitoring tools with uniform indicators will also help inform government at subnational and national levels as to whether they are achieving their targets towards decreasing pneumonia deaths.

## Additional material


Online Supplementary Document
