# Supplementary material for: Quality of care for children with acute respiratory infections in health facilities: a comparative analysis of assessment tools
Source: J Glob Health. 2022 Mar 26;12:10003. doi: 10.7189/jogh.12.10003 (PMC8942384; doi:10.7189/jogh.12.10003)

Supplementary appendix

Appendix S1 – Search strategies for systematic review on assessment tools for quality of care for children attending health facilities

MEDLINE Search strategy

#1	*"quality of health care"/ or *Quality Indicators, Health Care/
#2	(quality and health*).hw. and (mt or st or td or sn).fs.
#3	*"Delivery of Health Care"/
#4	1 and 3
#5	epidemiologic methods/ or data collection/ or datasets as topic/ or "surveys and questionnaires"/ or health care surveys/ or health surveys/ or exp population surveillance/
#6	Psychometrics/ or Interviews as Topic/ or reliability.tw.
#7	(tool or tools or observation*1).tw,kf.
#8	(evaluat* or measur* or assess* or data or monitor*).tw,kf.
#9	epidemiologic measurements/ or censuses/
#10	(1 or 2) and (5 or 6 or 7) and (8 or 9)
#11	*"quality of health care"/ or *"standard of care"/ or *culturally competent care/ or *health resources/ or exp *health services accessibility/ or *comparative effectiveness research/ or *"global burden of disease"/
#12	11 and (2 or 6) and (8 or 9)
#13	4 or 10 or 12
#14	*"quality of health care"/
#15	health facilities/ or hospital units/ or hospitals/
#16	(health-facilit* or (health* adj2 centre*) or (health* adj2 center*) or medical-center* or medical-centre*).tw,kf.
#17	(13 or 14) and (15 or 16)
#18	limit 17 to "all child (0 to 18 years)"
#19	limit 18 to (english language and yr="2008 -Current")

PubMed keyword search strategy

#1	Title/Abstract	(“quality of health care” OR “quality of health-care” OR “quality of healthcare” OR health-care-quality OR healthcare-quality OR “standard of health care” OR “standards of health care” OR “standard of health-care” OR “standards of health-care” OR “standard of healthcare” OR “standards of healthcare” OR health-care-standard* OR healthcare-standard*) AND
	Title/Abstract	(survey* OR questionnaire* OR psychometric* OR tool OR tools OR evaluat* OR measur* OR assess* OR monitor*) AND
	Title/Abstract	(Health-facilit* OR medical-facilit* OR hospital OR hospitals* OR health-centre* OR health-center* OR medical-center* OR medical-centre*) AND
#2	All fields	(newborn* OR new-born* OR baby OR babies OR neonat* OR neo-nat* OR infan* OR toddler* OR pre-schooler* OR preschooler* OR child OR children OR pediatric* OR paediatric*) AND (NOTNLM OR publisher[sb] OR inprocess[sb] OR pubmednotmedline[sb] OR indatareview[sb] OR pubstatusaheadofprint)
#3		#1 AND #2<br>Filters: Publication date from 01/01/2008 to 31/12/2020<br>Filters: English

Search results yielded for each database/ search site

	21/08/2020
MEDLINE	658
Global Health database	163
PubMed	255
International Journal for Quality in Healthcare	42
WHO Bulletin	62
WHO IRIS	27
World Bank	7
Hand-picked publications	9
Total	1223

Appendix S2. Prisma flow diagram for the selection of assessment tools

Appendix B: Prisma flow diagram for the selection of assessment tools

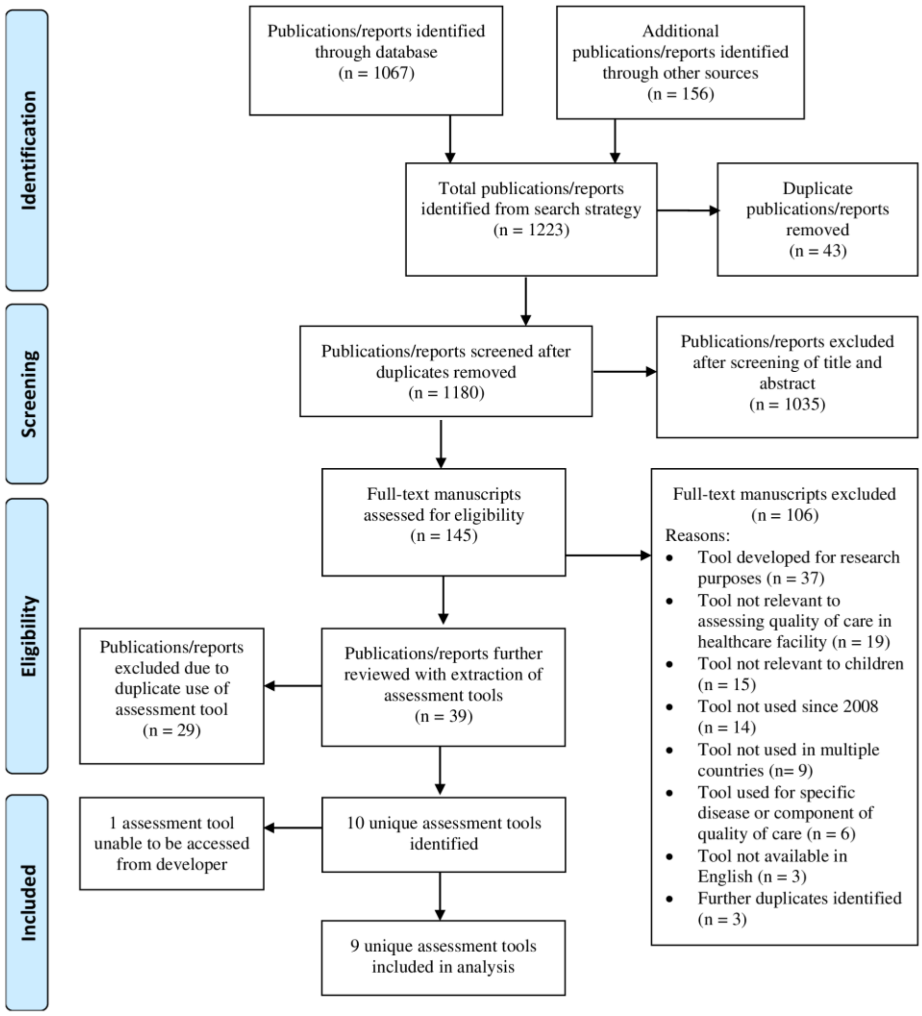

Supplement: Online Supplementary Document [file jogh-12-10003-s001.pdf]
